# External quality assessment of the entomological identification of triatomines in the network of public laboratories in Rondônia, Brazil

**DOI:** 10.1590/0037-8682-0211-2023

**Published:** 2023-09-22

**Authors:** Tatiana Oliveira Souza, João Paulo Sales Oliveira-Correia, Alda Lobato, Dayse da Silva Rocha, Cleber Galvão

**Affiliations:** 1 Instituto Oswaldo Cruz, Laboratório Nacional e Internacional de Referência em Taxonomia de Triatomíneos, Rio de Janeiro, RJ, Brasil.; 2 Secretária Estadual de Saúde de Rondônia, Laboratório Central de Saúde Pública, Porto Velho, RO, Brasil.

**Keywords:** Chagas disease, Vectors, Triatomines

## Abstract

**Background:**

An external quality assessment on the identification of triatomines within the laboratory network in the state of Rondônia.

**Methods:**

Seven laboratories participated in this evaluation. Each was provided with support materials and nine insects from the Hemiptera order for identification.

**Results:**

All samples were accurately identified at the species level. However, correct sex identification was achieved for only 79% of the samples. The most significant challenges were encountered in determining the sex of predators, phytophagous species, *Rhodnius robustus*, and *Rhodnius pictipes*.

**Conclusions:**

The identified shortcomings can inform enhancements in vector control programs for Chagas disease.

Triatomines, colloquially known as kissing bugs, are hematophagous insects that serve as vectors for *Trypanosoma cruzi* (Chagas, 1909), the protozoan responsible for Chagas disease. These vectors are prevalent across the country, with 64 species currently documented in Brazil and nine in the state of Rondônia. These include *Panstrongylus geniculatus* (Latreille, 1811), *Panstrongylus lignarius* (Walker, 1873), *Panstrongylus megistus* (Burmeister, 1835), *Panstrongylus rufotuberculatus* (Champion, 1899), *Eratyrus mucronatus* Stål, 1859, *Rhodnius montenegrensis* Rosa *et al*., 2012, *Rhodnius milesi* Carcavallo *et al.,* 2001, *Rhodnius robustus* Larrousse, 1927, and *Rhodnius pictipes* Stål, 1872^1^ ^,^ ^2^ ^,^ ^3^ ^,^ ^4^.

The accurate taxonomic identification of triatomines is essential for all subsequent studies, including entomological surveillance actions^5^. Consequently, the Laboratório Central de Saúde Pública of Rondônia State (LACEN/RO) conducted its inaugural *External Quality Assessment* (EQA) on triatomine identification within the state. The primary objective of this assessment was to analyze the quality control measures in place for identification procedures across its network of laboratories.

LACEN/RO serves as the state’s reference point, overseeing the quality control of laboratories conducting health surveillance-related diagnoses. In addition to this, it assumes the role of technical coordinator for other state entomology laboratories. These laboratories primarily focus on identifying disease vectors and etiological agents, including triatomines (Figure 1).

**FIGURE 1:**
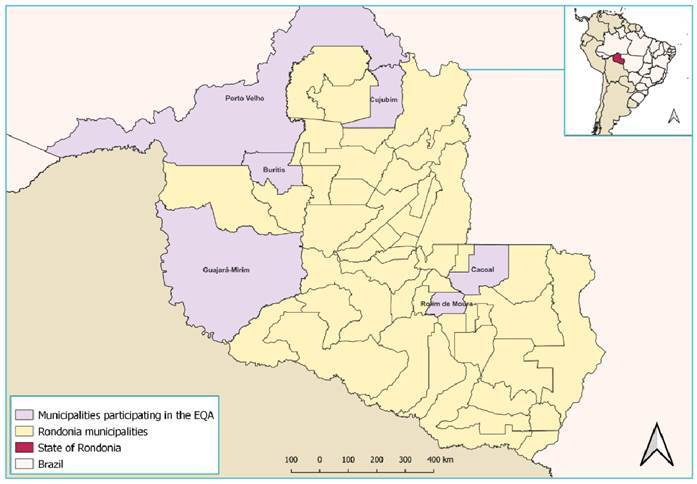
Map of the location of entomology laboratories, according to regional or municipal administrative management, Rondônia, 2023. Geodetic Reference System SIRGAS 2000. Geographical database acquired from the IBGE website (https://www.ibge.gov.br/) at Scale 1:1,000,000.

The aim of this study was to assess the capabilities of all laboratories in the state of Rondônia to screen and identify triatomines. The EQA began with the voluntary adherence of the participants who responded to the invitation letter sent by the provider, the Laboratório Nacional e Internacional de Referência em Taxonomia de Triatomíneos (LNIRTT), Oswaldo Cruz Institute, Fiocruz, Rio de Janeiro. The provider ensured impartiality, independence, and confidentiality in identifying the participants. The participating laboratories were provided with samples of each species, dichotomous keys, illustrated cards of the North region’s triatomines, and standardized forms for recording results. Each laboratory received a sample of each species, the identity of which was known solely to the provider, and results were recorded using a numerical system and a standardized form. The evaluation panel was composed of triatomine specimens bred in an insectarium at LNIRTT. The panel included species of epidemiological significance and wild species: *Eratyrus mucronatus*, *R. robustus*, *R. pictipes*, *P. lignarius*, *P. megistus*, *Triatoma maculata* (Erichson, 1848), *T. sordida* (Stål, 1859), along with a phytophagous insect (family Coreidae), and a predatory insect (family Reduviidae) (Figure 2). Of the selected triatomine species, five are found in the state of Rondônia, while two have not yet been recorded there. The species *P. geniculatus* and *R. montenegrensis* were excluded from the panel due to a shortage of available material. The specimens were affixed to Ethylene-Vinyl Acetate (EVA) sheets using an entomological pin, then packed in B-UN3373 type boxes for biological substances and dispatched by a company specializing in the transportation of biological material.

**FIGURE 2:**
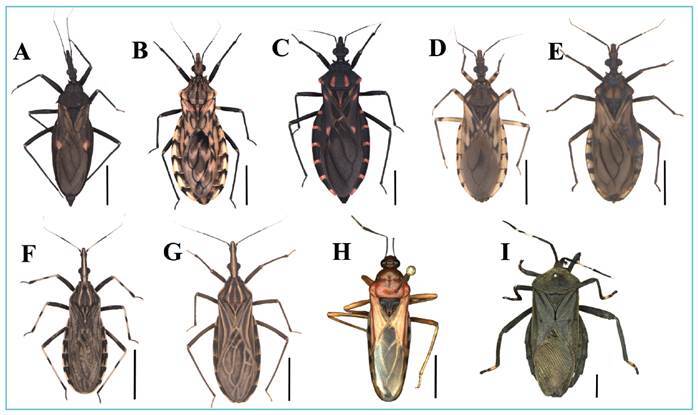
In dorsal view. **(A)** *Eratyrus mucronatus*, **(B)** *Panstrongylus lignarius*, **(C)** *Panstrongylus megistus*, **(D)** *Triatoma sordida*, **(E)** *Triatoma maculata*, **(F)** *Rhodnius pictipes*, **(G)** *Rhodnius robustus*, **(H)** Predator e **(I)** Phytophagous. Scale 5 mm.

Participants were directed to employ dichotomous keys as the primary tool for identification^1^. The ultimate deadline for transmitting the results to LACEN/RO was set at 15 days, commencing from the date the laboratory received the panel.

The variables analyzed included the condition of the specimen upon receipt (either perfect or damaged), the equipment used for examination (either a manual magnifying glass or a stereoscopic microscope), the sex of the identified specimen, and its specific taxonomic identification, where applicable. Data collection was conducted using a standardized response form, completed by the participating laboratory technician.

The laboratories’ species and sex identification results were juxtaposed with the provider's template and subsequently classified as either correct or incorrect. In the computation of the proportion of correct responses, unanswered queries were deemed incorrect.

Every laboratory utilized a stereoscopic microscope alon with a handheld magnifying glass. All participating laboratories confirmed receipt of the panel in a sealed condition and deemed it suitable for analysis. However, transportation damage to the legs and antennae was observed in 9.5% of the specimens. All laboratories adhered to the response submission deadline, which was also a factor in performance evaluation.

All participants accurately identified the triatomine species. The laboratories’ proportion of correct responses for species identification was 100% (63/63), compared to 79% (50/63) for differentiating between males and females. Table 1 displays the proportion of correct responses for the identification of triatomine species and sex. No laboratory achieved a perfect score (9/9) for sex differentiation. However, all species were correctly identified: *E. mucronatus*, phytophagous, *P. lignarius*, predator, *T. maculata*, *T. sordida*, *R. robustus,* and *R. pictipes.*

**TABLE 1:** Responses from laboratories in the identification of triatomines, according to sex (n=63), Rondônia, Brazil, 2022.

Reference samples	Laboratories						
	1	2	3	4	5	6	7
*Eratyrus mucronatus*	+	+	+	+	+	+	+
Phytophagous	+	+	+	+	-	-	-
Predator	+	-	+	+	-	+	-
*Panstrongylus lignarius*	+	+	+	+	+	-	+
*Panstrongylus megistus*	+	+	+	+	+	+	+
*Rhodnius pictipes*	+	-	+	-	+	+	+
*Rhodnius robustus*	-	-	-	-	+	+	-
*Triatoma maculata*	+	+	+	+	+	+	+
*Triatoma sordida*	+	+	+	+	+	+	+

(+) correct; (-) incorrect/no response.

The entomology laboratories involved in this study receive triatomines, captured by health agents working solely within households (intra and peridomicile), for identification. These insects may also be delivered by the public at Triatomine Information Points (PIT). However, these insects can sustain damage due to improper handling or during transportation. Despite this, our study found that damage to the antennae and legs did not hinder accurate species identification. The EQA plays a crucial role in ensuring the reliability of this identification process, thereby enhancing the quality of results that can be utilized in Chagas disease control programs.

The results indicated that the professionals evaluated possess the necessary technical proficiency to identify triatomine species in the State of Rondônia. It is important to emphasize that the EQA is not a definitive measure, as it reflects the technical quality of the professionals at the time of evaluation. Consequently, the EQA should be an ongoing process, keeping pace with changes in the laboratories, such as staff turnover and updates on the distribution and identification of new triatomine species. Generally, laboratories grapple with significant issues like employee turnover, resulting from temporary work contracts and retirements without immediate replacements, and inadequate equipment maintenance. These aspects were extensively discussed in a recent workshop hosted by the Health Surveillance Secretariat of the Ministry of Health^6^, and in two papers where the authors examined the understanding and knowledge of professionals involved in the entomological surveillance of Chagas disease and its vectors^7^ ^,^ ^8^_._

The challenge of taxonomic identification necessitates the cultivation of professional expertise. For instance, the species *R. robustus* and *R. prolixus* exhibit significant morphological similarities, potentially leading to identification errors. Such a situation has likely occurred previously, as evidenced by reports of *R. prolixus* presence in the state of Rondônia^9^. The inaugural EQA conducted for triatomine species identification in the State of Pernambuco underscored this issue, with species of the *Rhodnius* genus receiving the fewest correct identifications^10^.

LACEN/RO serves as the liaison, tasked with the upkeep of the EQA and the ongoing training of the seven affiliated entomology laboratories in the state of Rondônia. These laboratories bear the responsibility for the procurement and regular maintenance of microscopes, their involvement in the EQA program, and their participation in courses and training facilitated by LACEN/RO. The findings of the present study underscore the enduring necessity for training initiatives and the evaluation of diagnostic quality. Consequently, it is proposed that EQA activities be extended to other regions of Brazil to guarantee efficacy in the preventive and control measures against *T. cruzi* vectors.

ETHICAL CONSIDERATIONS

Each laboratory independently collected the samples, ensuring no conflict of interest.
