# Erratum

**DOI:** 10.1590/0037-8682-0221B-2021

**Published:** 2023-10-13

**Revista da Sociedade Brasileira de Medicina Tropical/Journal of the Brazilian Society of Tropical Medicine**

**Title:** External quality assessment of the entomological identification of triatomines in the network of public laboratories in Rondônia, Brazil 

 Vol.:56 | (e0211-2023) | 2023 - doi: https://doi.org/10.1590/0037-8682-0211-2023 - ACKNOWLEDMENTS 

 Page 4/4

 We offer our deepest thanks to the Central Public Health Laboratory, LACEN, Porto Velho, RO, Brazil, and the collaborators belonging to the network of laboratories in Rondônia. 

Should read:

We offer our deepest thanks to the Central Public Health Laboratory, LACEN, Porto Velho, RO, Brazil, and the collaborators belonging to the network of laboratories in Rondônia. We thank Dr. João Aristeu da Rosa and the entire team who provided access to photographs of various species from the Triatominae image bank at the Faculty of Pharmaceutical Sciences of Unesp-Araraquara - SP: https://www2.fcfar.unesp.br/#!/triatominae

